# Climate change worry, awareness, risk appraisal, and pro-environmental behaviors: Are these factors different for individuals with and without chronic diseases?

**DOI:** 10.1371/journal.pone.0325836

**Published:** 2025-06-13

**Authors:** Shiri Shinan-Altman, Yaira Hamama-Raz

**Affiliations:** 1 Louis and Gabi Weisfeld School of Social Work, Bar Ilan University, Ramat-Gan, Israel; 2 School of Social Work, Ariel University, Ariel, Israel; RAK Medical and Health Sciences University, UNITED ARAB EMIRATES

## Abstract

**Background:**

Climate change poses significant risks to human health, particularly exacerbating conditions for individuals with chronic diseases. This study aimed to examine differences in climate change awareness, risk appraisal, pro-environmental behaviors (PEBs), and climate change worry between individuals with and without chronic diseases, and to investigate their interrelationships.

**Methods:**

A cross-sectional survey design was employed, using convenience sampling. Participants included 405 Israeli adults (146 with chronic diseases, and 259 without) who completed validated self-report questionnaires assessing climate change awareness, risk appraisal, PEBs, and worry. Data analyses included descriptive statistics, Pearson correlations, and multivariate analysis of covariance, using SPSS version 29. Moderated serial mediation analysis was conducted using Hayes’ PROCESS macro (model 92) with 5,000 bootstrap samples.

**Results:**

Participants with chronic diseases reported significantly higher levels of climate change awareness, F(1, 400)=5.88, *p* = .016; risk appraisal, F(1, 400)=12.68, *p* < .001; PEBs, F(1, 400)=4.00, *p* = .046; and worry, F(1, 400)=6.81, *p* = .009, than did participants without chronic diseases. The moderated serial mediation model was significant (effect = 0.02, SE = 0.01, 95%CI [0.001, 0.04]), explaining 44% of the variance in climate change worry. Awareness positively predicted risk appraisal (B = 0.33, *p* < .001), which in turn predicted both PEBs (B = 0.23, *p* < .001) and worry (B = 0.29, *p* < .001). The indirect pathway from awareness to worry via PEBs was significant only among participants with chronic diseases (B = 0.04, SE = 0.02, 95%CI [0.01, 0.10]). Similarly, the complete serial mediation path—from awareness to risk appraisal, to PEBs, and finally to worry—was significant for participants with chronic diseases (B = 0.02, SE = 0.01, 95%CI [0.01, 0.05]) but not for participants without chronic diseases.

**Conclusions:**

The results emphasize the need for targeted communication strategies and policy initiatives that address the specific vulnerabilities and behaviors of chronically ill populations. Future research should utilize longitudinal approaches and objective assessments to deepen our understanding of these dynamics and inform effective interventions.

## Introduction

Climate change represents a significant global issue affecting human health and the environment. Increased air pollution, higher temperatures, and frequent extreme weather events negatively influence public health, contributing to various health problems, including heat-related diseases, respiratory diseases, infectious diseases, and psychological distress [1–7]. These changes particularly threaten vulnerable populations, such as individuals living with chronic health conditions, who are more susceptible to environmental hazards and health complications.

Raising public awareness and encouraging pro-environmental behaviors (PEBs) are vital strategies for mitigating climate change effects. Climate change awareness involves recognizing the seriousness of climate change and understanding the related risks [8–10]. Higher levels of awareness motivate people to adopt environmentally friendly behaviors, such as recycling, conserving energy, or supporting sustainable products [11]. Promoting these behaviors contributes significantly to global efforts in combating climate change [12,13].

Individuals with chronic diseases (such as cardiovascular or respiratory conditions) are disproportionately affected by climate change. For example, increased heat can worsen cardiovascular symptoms, and poor air quality from wildfires or pollution significantly impacts those with asthma or chronic obstructive pulmonary disease (COPD) [2,4]. Additionally, climate-driven increases in infectious diseases pose heightened risks to individuals with compromised immune systems or existing chronic conditions [5]. Given these vulnerabilities, understanding the relationship between climate change and chronic disease populations becomes critical.

Climate change worry, characterized by anxiety and concern regarding climate change impacts, strongly influences individual behaviors. Moderate worry can motivate positive actions and support for climate-related policies, whereas excessive worry may cause feelings of powerlessness or resignation [12,14–16]. Awareness and perceptions of climate risk directly shape these psychological responses and associated actions [17–19]. Risk appraisal refers to individuals’ subjective evaluation of environmental hazards (e.g., heatwaves, air pollution, droughts) and their potential harm [20,21]. Pro-environmental behaviors represent active coping strategies aimed at reducing environmental impacts and mitigating climate change.

**Fig 1:**
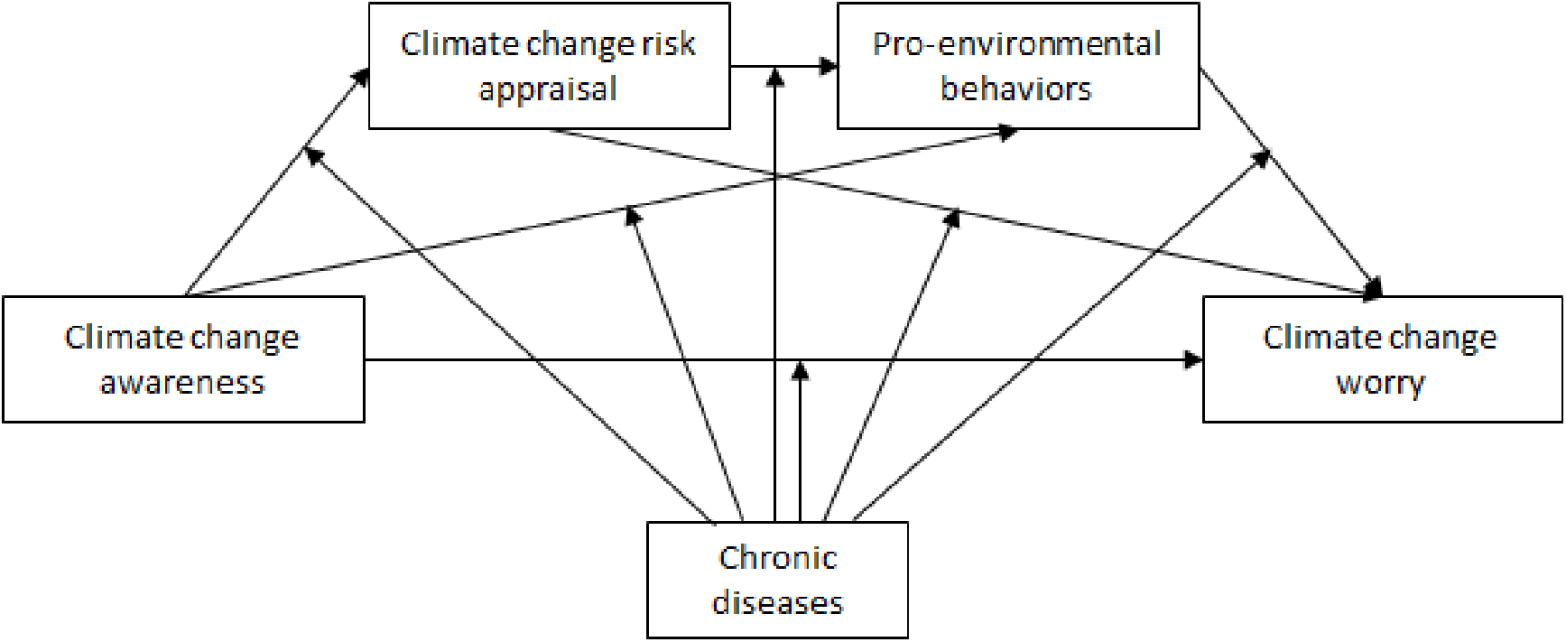
The study model.

Drawing from Lazarus and Folkman’s transactional theory of stress and coping [22], in the current study we explored how climate change awareness was associated to climate change worry through the mediators of climate risk appraisal and PEBs (Fig 1). According to this framework, climate change awareness influences risk appraisal, which subsequently predicts both PEBs and climate change worry. We expected chronic disease status to moderate these relationships, with participants who experienced chronic diseases exhibiting heightened sensitivity to climate change-related threats and showing stronger psychological and behavioral responses.

Based on this framework, we posited the following hypotheses:

H1. Individuals with a chronic disease would report higher levels of climate change awareness, climate change risk appraisal, PEBs, and climate change worry than would participants without a chronic disease.
H2. Climate change awareness, climate change risk appraisal, and PEBs would be positively associated with climate change worry.
H3. Climate change risk appraisal and PEBs would mediate the association between climate change awareness and climate change worry.
H4. Having a chronic disease would moderate the mediated associations between climate change awareness, climate change risk appraisal, PEBs, and climate change worry, such that the associations would be stronger for participants who had a chronic disease.

By specifically examining whether having a chronic disease moderates the abovementioned psychological and behavioral responses, the current study makes a significant contribution to the existing research. Understanding these differences can inform targeted global and local interventions, enhance policy development, and improve climate communication strategies specifically tailored to vulnerable populations.

## Materials and methods

Ethics approval was received from the Ethics Committee of the first author university (approval No. 032403). Participants provided their consent to participate in the study by signing a consent form. This study was reported according to the Strengthening the Reporting of Observational Studies in Epidemiology (STROBE).

### Procedure

In this study, we used an Internet panel of Israelis (iPanel) that aligns with Israel’s Bureau of Statistics in key sociodemographic factors including age, gender, and marital status, and represents the general population [23]. This professional survey company in Israel provides monetary incentives to respondents – a common practice to encourage participation and enhance representativeness, similar to well-established international survey companies such as Qualtrics and Survey Junkie in the USA. The study was approved by the first author’s university’s institutional review board (IRB; approval No. 032403). Data collection was conducted during the last two weeks of March 2024. Each participant was required to sign an electronic informed written consent form before accessing the questionnaire. All participants’ responses were recorded and organized in an Excel file provided as S1 File (Supporting Information).

### Measurements

Participants completed the following self-report questionnaires.

***Climate change worry*** was assessed via Stewart’s [14] climate change worry scale, which includes ten items (e.g., “I worry about climate change more than other people do”) on a 5-point Likert scale ranging from 1 (*never*) to 5 (*often*). A mean score was calculated; a higher score indicated higher levels of climate change worry. In the current study, Cronbach’s alpha for internal consistency was 0.93.

***Climate change awareness*** was assessed via a single question drawn from the research of Lee and colleagues [24]. The question was: “How much do you know about global warming or climate change?” The available answers were: 1 (*I have never heard of it*), 2 (*I know something about it*), and 3 (*I know a lot about the effects of climate change)*.

***Risk appraisal of the effects of climate change*** was assessed via six items pertaining to harm and threat appraisal of climate change [20]. On a 5-point Likert scale ranging from 1 (*strongly agree*) to 5 (*strongly disagree*), participants were asked to rate how much they agreed or disagreed with each statement (e.g., “My health has worsened because of the pollution in everyday life”). A mean score was calculated; a higher score indicated higher levels of climate change risk appraisal. In the current study, Cronbach’s alpha for internal consistency was 0.71.

***Pro-environmental behaviors*** *(PEBs)* were assessed via the Homburg and Stolberg [20] questionnaire, which initially included eight items. However, we removed one item due to a negative correlation with the others. The revised questionnaire comprised seven items (e.g., “I take part in events run by environmental organizations, such as setting up nest boxes or toad fences” or “I take part in litter clean-ups”), each rated on a 5-point Likert scale ranging from 1 (*strongly agree*) to 5 (*strongly disagree*). A mean score was calculated; a higher score indicated higher PEB levels. In the current study, Cronbach’s alpha for internal consistency was 0.62.

*Sociodemographic variables* included gender, age, area of residence, years of education, marital status (married; divorced; widowed; single), number of children, employment status (full-time; part-time; self-employed; unemployed; pensioner; stay-at-home-parent), religiosity (secular, traditional, religious/Orthodox). In addition, participants were asked to note whether they had been diagnosed with a chronic disease (yes/no), the type of chronic disease they had (if yes), and how long they had been coping with it (i.e., when they had first been diagnosed).

### Statistical analysis

Data were analyzed with SPSS ver. 29. As data were collected by a survey company, there were no missing data. Descriptive statistics were used for participants’ demographic characteristics and for the study variables. Internal consistencies were calculated with Cronbach’s α, and Pearson correlations were calculated among the study variables. Associations between the demographic variables and the study variables were examined with Pearson correlations and *t*-tests, to identify potential covariates. Level of education did not deviate from normal distribution (skewness = −0.28, SE = 0.12) and was thus regarded as a continuous variable. Differences in the study variables, in accordance with the presence of a chronic disease, were examined with multivariate analysis of covariance (MANCOVA), controlling for gender, age, and level of education. The study model was assessed with the PROCESS procedure [25], using model 92 for moderated serial mediation, with 5,000 bootstrap samples and 95% confidence intervals. Variables were standardized, and significant interactions were plotted with simple slopes.

## Results

### Participants

Inclusion criteria included being at least 18 years old and fluent in Hebrew. Exclusion criteria included people with cognitive impairments or those unable to provide informed consent. The sample size was calculated using G*Power software [26,27]. For an analysis of covariance with two groups and several covariates, with a moderate-low effect size f = 0.15, α = .05, and power = 0.80, the required sample size would be N = 351. This sample size provides a power level of 0.98 for a multiple regression model with up to 15 predictors, α = .05, and a moderate-low effect size f^2^ = 0.10. The current study comprised 405 people, above and beyond the required 351.

Of the 405 participants, 209 (51.6%) were men and 196 (48.4%) were women, aged 19–70 years (mean = 44.17, SD = 14.58). Fifty-eight percent of the sample were married or in a relationship (*n* = 235), and 67.9% (*n* = 275) had children. Two-hundred-and-fifty-seven (63.4%) had more than 12 years of education. Regarding their religiosity level, 215 (53.1%) were secular, 127 (31.3%) were traditional, and 63 (15.6%) were religious or Orthodox. With regard to health status, about a third of the participants (*n* = 146, 36%) reported coping with a chronic disease. Common chronic diseases were allergies (*n* = 66, 16.3%), high blood pressure (*n* = 56, 13.8%), asthma (n = 26, 6.4%), diabetes (*n* = 25, 6.2%), and gastrointestinal diseases (*n* = 18, 4.4%). Table 1 presents sample demographics.

**Table 1:** Demographic characteristics (N = 405).

Variable	Categories	Values
Age, Mean (SD), range		44.17 (14.58), 19-70
Gender, n(%)	Male	209 (51.6)
	Female	196 (48.4)
Marital status, n(%)	Single	122 (30.1)
	Married, in a relationship	235 (58.0)
	Divorced, widowed, other	48 (11.9)
Number of persons at home, Mean (SD), range		3.53 (1.62), 1-11
Children, n(%)	Yes	275 (67.9)
Number of children, Mean (SD), range		1.21 (1.19), 0-6
Level of education, n(%)	Up to high school, high school	148 (36.5)
	Professional certification	69 (17.0)
	Higher education	188 (46.4)
Income, n(%)	Below average	134 (34.5)
	Average	92 (23.7)
	Above average	162 (41.8)
Religiosity, n(%)	Secular	215 (53.1)
	Traditional	127 (31.3)
	Religious/Orthodox	63 (15.6)
Chronic diseases, n(%)	Yes	146 (36.0)

SD = standard deviation.

In order to examine possible differences between participants with a chronic disease compared to participants without a chronic disease, MANCOVA was conducted. Differences in the study variables, by the presence of a chronic disease, were found to be significant, with small effect sizes (Table 2). Levels of awareness, climate change risk appraisal, PEBs, and climate change worry were all higher among participants with a chronic disease than among participants without one. Accordingly, Hypothesis 1 was accepted.

**Table 2:** Means, standard deviations, and F values for the study variables among people with and without a chronic disease.

Variables	Chronic disease (N = 146)	No chronic disease (N = 259)	F (1, 400) (*p*)
	Mean (SD)	Mean (SD)	(η^2^)
Climate change awareness	3.65 (0.94)	3.42 (0.92)	5.88 (*p* = .016) (η^2^ = .014)
Climate change risk appraisal	3.15 (0.66)	2.89 (0.64)	12.68 (*p* < .001) (η^2^ = .031)
Pro-environmental behaviors	2.85 (0.61)	2.67 (0.64)	4.00 (*p* = .046) (η^2^ = .010)
Climate change worry	2.42 (0.83)	2.19 (0.76)	6.81 (*p* = .009) (η^2^ = .017)

SD, standard deviation.

In order to examine Hypothesis 2, Pearson correlations were calculated among the study variables (Table 3). Findings revealed significant positive correlations among all the study variables. That is, higher climate change awareness, higher climate change risk appraisal, and higher PEBs were positively associated with one another, and all were positively associated with higher climate change worry. Accordingly, Hypothesis 2 was accepted.

**Table 3:** Means, standard deviations, and Pearson correlations for the study variables (N = 405).

Variables	Mean (SD)	Chronic diseases	Awareness	Climate change risk appraisal	Pro-environmental behavior	Climate change worry
Chronic disease (yes)	0.36 (0.48)	–				
Climate change awareness	3.50 (0.93)	.12*	–			
Climate change risk appraisal	2.99 (0.66)	.19***	.36***	–		
Pro-environmental behaviors	2.74 (0.63)	.14**	.28***	.32***	–	
Climate change worry	2.27 (0.79)	.14**	.54***	.52***	.34***	–

*p < .05, **p < .01, ***p < .001 Note. Ranges: 1–5. SD, standard deviation.

In addition, associations with demographic variables were examined in order to identify potential covariates. Age was positively associated with PEBs (r = .17, *p* < .001), and level of education (ranging from elementary school to a graduate degree of an MA or PhD with skewness = −0.28, SE = 0.12) was positively associated with climate change awareness (r = .15, *p* = .003). Other associations, as well as associations with other demographic variables (gender, marital status, having children, income level, and religiosity), were not significant. In light of these associations, age and level of education were controlled for in further analyses. Gender was controlled for as well, despite its non-significant association with the study’s variables, due to its potential relevance in general, and its relevance to chronic diseases in particular.

The moderated serial mediation model was found to be significant (effect = 0.02, SE = 0.01, 95%CI = 0.001, 0.04), with 44% of the variance in climate change worry being explained by it. Climate change awareness was positively associated with climate change risk appraisal (B = 0.33, SE = 0.06, *p* < .001, 95%CI = 0.21, 0.44), regardless of whether the participant had a chronic disease (B = 0.04, SE = 0.10, *p* = .663, 95%CI = −0.15, 0.23). Further, climate change risk appraisal was positively associated with PEBs (B = 0.23, SE = 0.06, *p* < .001, 95%CI = 0.11, 0.36), regardless of whether the participant had a chronic disease (B = 0.01, SE = 0.10, *p* = .953, 95%CI = −0.20, 0.21). In addition, climate change awareness was positively associated with PEBs (B = 0.21, SE = 0.06, *p* < .001, 95%CI = 0.09, 0.34), regardless of whether the participant had a chronic disease (B = −0.04, SE = 0.10, *p* = .712, 95%CI = −0.13, 0.28). Thus, the mediation of climate change risk appraisal, between climate change awareness and PEBs, was supported beyond having a chronic disease (effect = 0.05, SE = 0.01, 95%CI = 0.03, 0.08). Moreover, climate change risk appraisal was positively associated with climate change worry (B = 0.29, SE = 0.04, *p* < .001, 95%CI = 0.21, 0.38), regardless of whether the participant had a chronic disease (B = −0.06, SE = 0.07, *p* = .372, 95%CI = −0.20, 0.07). Thus, the mediation of climate change risk appraisal, between climate change awareness and climate change worry, was supported beyond having a chronic disease (effect = 0.11, SE = 0.02, 95%CI = 0.07, 0.15). Accordingly, Hypothesis 3 was accepted.

Only one significant interaction was found between having a chronic disease and PEBs (B = 0.21, SE = 0.07, *p* = .002, 95%CI = 0.08, 0.35). Namely, the mediated association between climate change awareness, PEBs, and climate change worry was moderated by the presence of a chronic disease, such that this association was significant for participants with a chronic disease (B = 0.04, SE = 0.02, 95%CI = 0.01, 0.10), and not significant for participants without a chronic disease (B = 0.01, SE = 0.01, 95%CI = −0.01, 0.03). Specifically, the presence of a chronic disease strengthened the indirect relationship, indicating that participants with chronic diseases who were more aware of climate change engaged in higher levels of PEBs which, in turn, increased their climate change worry. In contrast, participants without chronic diseases did not demonstrate this significant indirect pathway.

**Fig 2:**
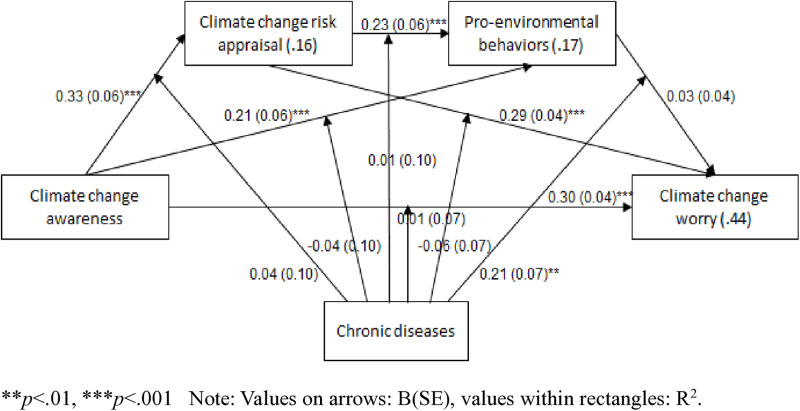
The moderated serial mediation model for climate change awareness, climate change risk appraisal, pro-environmental behaviors, and climate chage worry, by the presence of a chronic disease.

**Fig 3:**
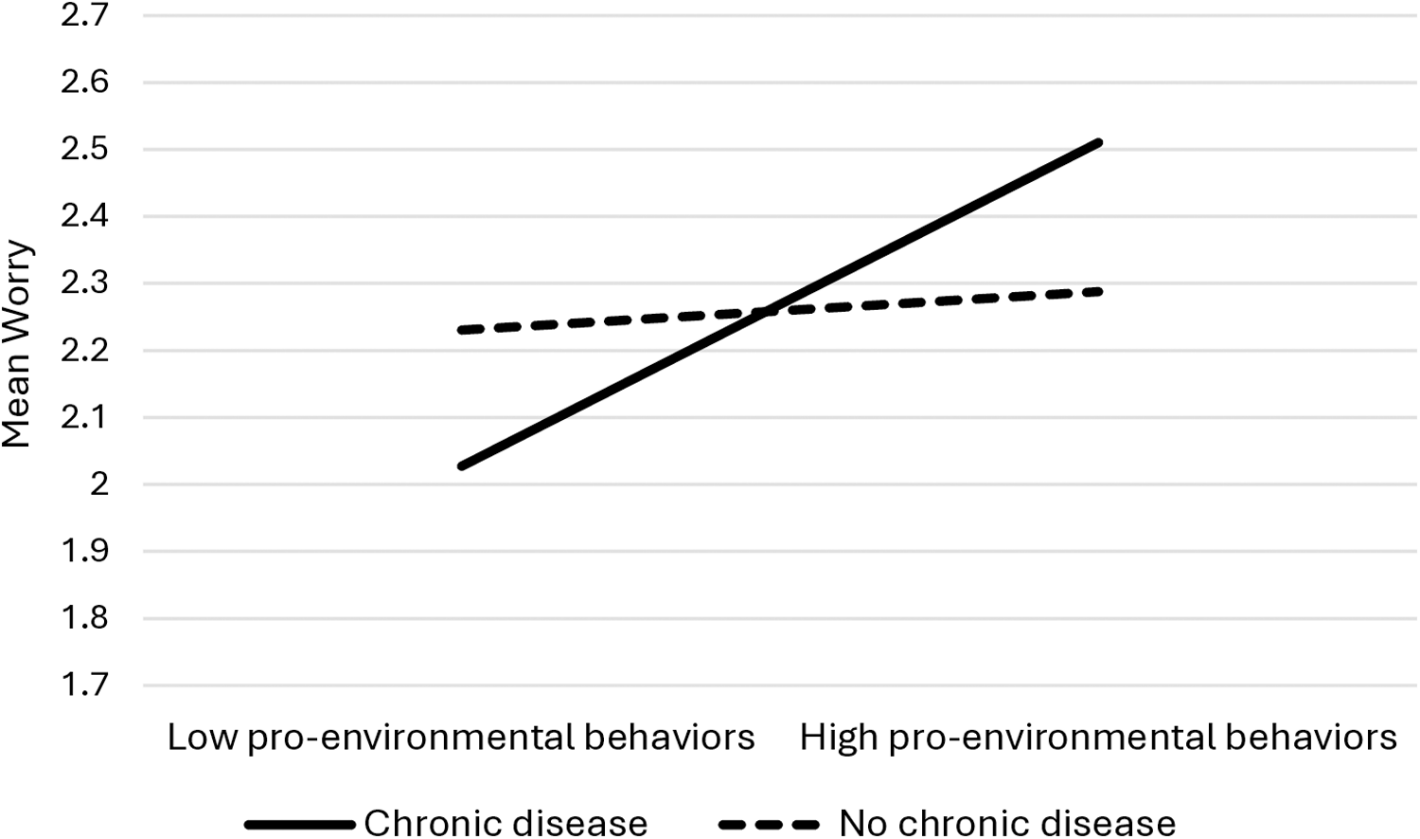
Pro-environmental behaviors with climate change worry, by the presence of a chronic disease.

**Fig 4:**
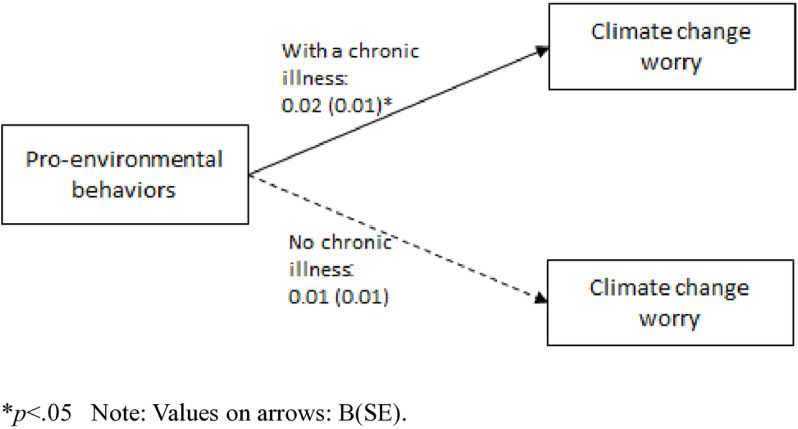
The direct association between pro-environmental behaviors and climate change worry, by the presence of a chronic disease.

Similarly, the full serial mediation model – in which climate change awareness was associated with climate change risk appraisal, thereby being associated with PEBs, and then with climate change worry – was moderated by having a chronic disease, such that it was significant for participants with a chronic disease (B = 0.02, SE = 0.01, 95%CI = 0.01, 0.05), but not significant for participants without a chronic disease (B = 0.01, SE = 0.01, 95%CI = −0.01, 0.01). Having a chronic disease therefore amplified the indirect effects across the serial mediation model, such that increased climate change awareness led to greater risk appraisal, higher engagement in PEBs, and ultimately increased climate change worry only among participants with a chronic disease. These moderation effects were illustrated using simple slopes. Accordingly, Hypothesis 4 was partially accepted (Figs 2–4).

*In sum*, climate change risk appraisal mediated the relationship between climate change awareness and both PEBs and climate change worry. That is, awareness led to greater risk appraisal, which in turn led to more PEBs and worry. In addition, having a chronic disease moderated the indirect path from awareness to worry through PEBs. That is, the relationship was significant for participants with a chronic disease, but not for participants without one.

## Discussion

The aim of the current study was to examine differences in climate change awareness, risk appraisal, PEBs, and climate change worry between individuals with and without chronic diseases, and to investigate their interrelationships. Our findings revealed that levels of climate change awareness, climate change risk appraisal, PEBs, and climate change worry were higher among participants with a chronic disease than among those without a chronic disease, highlighting this group’s heightened sensitivity to climate-related factors. Moreover, the indirect link between climate change awareness and climate change worry via PEBs was moderated by having a chronic disease. These findings suggest that having a chronic disease significantly affects how individuals perceive and react to environmental issues.

Specifically, in line with the higher levels of climate change-related factors that were reported among the participants with chronic diseases in the current study, previous research has indicated that individuals who perceive themselves as vulnerable due to health or other factors are more attentive to related risks. For example, a study by the Environmental Protection Agency [2] highlighted that individuals with chronic respiratory diseases may experience heightened sensitivity to air quality issues exacerbated by climate change, potentially leading to increased awareness and concern. This finding aligns with findings from a previous study suggesting that personal vulnerability enhanced environmental risk appraisal [20].

Moreover, the significant positive correlations we found in the current study between climate change awareness, risk appraisal, PEBs, and climate change worry echo findings from a previous study conducted among 302 residents of a coastal community in the Northeastern U.S. In that study, it was revealed that exposure to flood risk information significantly influenced threat perceptions and coping efficacy (i.e., similar to the findings of our study, increased climate change awareness was found to correlate positively with higher risk appraisal [28]). This pattern is consistent with recent findings demonstrating that direct or indirect exposure to climate change-related events contributes to heightened risk appraisal and emotional responses [16]. This finding also aligns with the premise that cognitive factors play a pivotal role in the processing of environmental threats. Furthermore, the positive correlation between risk appraisal and engagement in PEBs resonates with the Protection Motivation Theory [29], suggesting that individuals are more likely to adopt protective behaviors when they perceive the threat as significant. Our study supports this notion, indicating that individuals who appraise climate risks more seriously are more inclined to engage in behaviors aimed at mitigating these risks. Additionally, our findings indicated that increased awareness of environmental risks was associated with engagement in PEBs, which was consequently associated with more climate change worry. Indeed, active involvement in environmental issues has been found to deepen individuals’ understanding of the severity of climate change, thereby escalating concerns [15,30].

The findings from the serial mediation model underscore a significant linkage between risk appraisal and PEBs in the association between climate change awareness and climate change worry. The model reveals that increased awareness of climate change significantly enhances the perception of climate-related risks, and this perception, in turn, is associated positively with both PEBs and climate change worry. This finding aligns with findings from a Norwegian study demonstrating that exposure to information on global warming correlated with heightened perceived threats as a result of climate change and elevated concerns among the public [31]. This enhanced risk perception drives behavioral change and emotional responses, illustrating a pathway that is sustained irrespective of individual health conditions such as chronic diseases.

Additionally, the role of risk appraisal as a mediator in the link between climate change awareness and climate change worry, and between PEBs and climate change worry, highlights the crucial role of cognitively processing risks in terms of driving both action and emotional responses to climate change. Drawing on the transactional theory of stress and coping [22], which posits that individuals continuously assess environmental stimuli to manage stress, risk appraisal seems to serve as a crucial mediator between climate change awareness and worry.

This conceptualization underscores how understanding and interpreting climate risks are vital in motivating appropriate responses and managing associated emotional impacts.

The significant moderating role of chronic disease in the link between engagement in PEBs and climate change worry reveals a relationship in which health status is associated with climate-related behaviors and concerns. The moderated effect indicates that individuals with chronic diseases are likely more attuned to the implications of environmental degradation on their health, leading to both increased engagement in PEBs and heightened worry about climate change. This finding aligns with findings from a previous study which demonstrated that individuals with health vulnerabilities often perceive environmental threats as more immediate and severe, thus potentially driving stronger engagement in behaviors aimed at mitigating such risks [21]. Furthermore, the moderation by chronic disease status in the serial mediation model between climate change awareness, risk appraisal, PEBs, and climate change worry emphasizes the role of personal health in environmental perception and action.

Several limitations should be noted. First, the study’s cross-sectional design hinders our ability to draw causal inferences between climate change awareness, risk appraisal, PEBs, and climate change worry. Future research would benefit from the use of longitudinal or experimental designs to establish causality. Second, the reliance on self-report measures, which are subject to various response biases, may not always accurately reflect study participants’ actual experiences or behaviors, potentially due to recall errors, misunderstanding of questions, or social desirability biases. Incorporating objective measures such as physiological stress markers or direct behavioral tracking in future studies could help mitigate these biases. Additionally, the sample, drawn from an Internet panel, may not have fully represented the wider population or individuals with chronic diseases. Going forward, researchers would do well to recruit larger samples with a more representative number of individuals with chronic diseases, thus allowing for the potential generalization of results. Lastly, the focus on a single country also limits the generalizability of the results. Israel has unique sociocultural, political, and environmental characteristics, as well as distinct climate conditions characterized by frequent heatwaves, droughts, and limited water resources. These specific conditions might uniquely influence climate change perceptions, behaviors, and concerns, limiting the generalizability of the findings to countries or regions with different climate profiles. Future studies should compare these results with populations experiencing varied climatic conditions to better understand context-specific influences.

## Conclusions

This study underscores the intricate interplay between having a chronic disease and climate change-related factors, revealing that individuals with chronic conditions exhibit heightened climate change awareness and responsiveness to environmental challenges. These findings suggest that having a chronic disease influences environmental perceptions and behaviors and thus emphasizes the importance of incorporating health status in climate policy discussions. In addition, the significant positive correlations found between climate change awareness, risk appraisal, PEBs, and climate change worry highlight the interconnected nature of cognitive, emotional, and behavioral responses to environmental threats. These insights provide critical implications for policymakers: Health considerations should not be treated as separate from climate action, but rather as integral to it.

Policymakers are encouraged to adopt a health-informed approach to climate interventions, recognizing that individuals with chronic diseases—due to their heightened awareness and sensitivity—can serve as key stakeholders and agents of change. In alignment with this perspective, the WHO [32] has emphasized the importance of health-centered climate communication to enhance relevance and drive behavior change, particularly among vulnerable populations. Global initiatives, such as the Lancet Countdown on Health and Climate Change [33], similarly advocate for integrating health considerations into climate policies to address both environmental and health-related risks. As we move forward, it is crucial that policymakers incorporate these differential impacts into interventions aimed at strengthening community resilience. Such interventions could include targeted educational programs for individuals with chronic diseases, healthcare-based initiatives to promote environmental engagement, and the integration of climate adaptation strategies into chronic disease management. Ensuring access to resilient healthcare infrastructure is also essential for protecting medically vulnerable populations during climate events. International collaborations and global climate frameworks, such as those guided by the United Nations Framework Convention on Climate Change (UNFCCC), should prioritize the unique needs of individuals with chronic diseases. These findings extend beyond the Israeli context, highlighting the global potential for tailored climate communication and health interventions to enhance engagement, reduce health disparities, and promote equitable and inclusive climate resilience.

## Supporting information

S1 File Excel file containing the raw data collected for the study. (XLSX)
